# Bacterial Loads Measured by the Xpert MTB/RIF Assay as Markers of Culture Conversion and Bacteriological Cure in Pulmonary TB

**DOI:** 10.1371/journal.pone.0160062

**Published:** 2016-08-10

**Authors:** Shubhada Shenai, Katharina Ronacher, Stefanus Malherbe, Kim Stanley, Magdalena Kriel, Jill Winter, Thomas Peppard, Charles E. Barry, Jing Wang, Lori E. Dodd, Laura E. Via, Clifton E. Barry, Gerhard Walzl, David Alland

**Affiliations:** 1 Division of Infectious Diseases, Rutgers New Jersey Medical School, ^Rutgers Biomedical & Health Sciences (Formerly UMDNJ), 185 South Orange Avenue, Newark, New Jersey, United States of America; 2 DST/NRF Centre of Excellence for Biomedical TB Research and MRC Centre for TB Research, Division of Molecular Biology & Human Genetics, Department of Biomedical Sciences, Faculty of Medicine & Health Sciences, Stellenbosch University, Cape Town, South Africa; 3 Catalysis Foundation for Health, Emeryville, California, United States of America; 4 Certara, LP, on contract to the Bill and Melinda Gates Foundation, Greater Detroit Area, United States of America; 5 Brown University, Providence Rhode Islands, United States of America; 6 Clinical Research Directorate/Clinical Monitoring Research Program, Leidos Biomedical Research, Inc., NCI Campus at Frederick, Frederick, Maryland, United States of America; 7 Biostatistics Research Branch, NIAID, NIH, Bethesda, Maryland, United States of America; 8 Tuberculosis Research Section, Laboratory of Clinical Infectious Diseases, NIAID, National Institutes of Health, Bethesda, Maryland, United States of America; 9 Institute of Infectious Disease and Molecular Medicine, and the Department of Clinical Laboratory Sciences, Faculty of Health Sciences, University of Cape Town, Rondebosch 7701, South Africa; Institut de Pharmacologie et de Biologie Structurale, FRANCE

## Abstract

**Introduction:**

Biomarkers are needed to monitor tuberculosis (TB) treatment and predict treatment outcomes. We evaluated the Xpert MTB/RIF (Xpert) assay as a biomarker for TB treatment during and at the end of the 24 weeks therapy.

**Methods:**

Sputum from 108 HIV-negative, culture-positive pulmonary TB patients was analyzed using Xpert at time points before and during anti-TB therapy. Results were compared against culture. Direct Xpert cycle-threshold (Ct), a change in the Ct (delta Ct), or a novel “percent closing of baseline Ct deficit” (percent closing) were evaluated as classifiers of same-day and end-of-treatment culture and therapeutic outcomes.

**Results:**

Xpert was positive in 29/95 (30.5%) of subjects at week 24; and positive one year after treatment in 8/64 (12.5%) successfully-treated patients who remained free of tuberculosis. We identified a relationship between initial bacterial load measured by baseline Xpert Ct and time to culture conversion (hazard ratio 1.06, p = 0.0023), and to the likelihood of being among the 8 treatment failures at week 24 (AUC = 72.8%). Xpert Ct was even more strongly associated with culture conversion on the day the test was performed with AUCs 96.7%, 99.2%, 86.0% and 90.2%, at Day 7, Week 4, 8 and 24, respectively. Compared to baseline Ct measures alone, a combined measure of baseline Ct plus either Delta Ct or percent closing improved the classification of treatment failure status to a 75% sensitivity and 88.9% specificity.

**Conclusions:**

Genome loads measured by Xpert provide a potentially-useful biomarker for classifying same day culture status and predicting response to therapy.

## Introduction

There are few reliable biomarkers to monitor the efficacy of tuberculosis (TB) treatment and predict treatment outcomes. Markers of elevated bacterial load such as the detection of acid fast bacilli (AFB) in sputum smears, or identification of lung cavities on chest X ray have been associated with worse treatment outcomes **[1, 2]**. However, the predictive values of these indicators are relatively weak and are often not useful for individualizing therapy. Two-month culture conversion is the most widely accepted marker for assessing treatment efficacy. Indeed, one meta-regression analysis used regimen duration and rate of two month culture status to predict relapse rates in the various arms of REMOX, OFLOTUB and RIFAQUIN at an R2 = 0.86 [3]. While culture may have a value as a trial-level prognostic factor in the context of developing novel regimens, other studies **[1, 4]**, including a systematic review and meta-analysis [5], report that two-month culture conversion had very poor sensitivity and specificity for predicting treatment failure and relapse in individual patients, including a recent analysis of the REMOX trial [6]. Furthermore, culture facilities are often unavailable to populations with high burdens of TB [7, 8] and if available, cultures must be continued for at least 42 days before they can be identified as negative. The long delay associated with a culture result can also contribute to the difficulty of using cultures to identify patients who have failed conventional therapy or interrupted their treatment. **[5, 9]**. This may be a particularly difficult problem in patients suspected of having drug resistant TB. All these patients may require additional months of treatment. The paucity of treatment response biomarkers also complicates clinical trials of new tuberculosis therapies **[5, 10]**.

The GeneXpert MTB/RIF (Xpert) assay is an automated, rapid, near-patient real time PCR assay that simultaneously detects *Mycobacterium tuberculosis* and Rifampicin (RIF) resistance**[11–14]**. The assay also has a quantitative function, suggesting that it could be used to measure bacterial load and perhaps predict treatment response **[13]**. However, several recent studies have suggested that the Xpert assay performs poorly for this purpose **[15–17]**. This is perhaps due to the fact that DNA from dead *M*. *tuberculosis* organisms is likely to persist in a TB patient’s sputum for some time, making it difficult for the Xpert assay to distinguish between live and successfully killed organisms **[18]**. However, prior studies have not appeared to make full use of the quantitative capabilities of the Xpert assay to stratify patients by likelihood of culture conversion. Nor have these studies fully explored the ability of serial Xpert tests to demonstrate a treatment effect by detecting a drop in the amount of *M*. *tuberculosis* DNA present in sputum samples. Finally, these prior studies did not look at whether Xpert results could predict treatment outcomes, which is clearly the most important potential use of a TB treatment biomarker. Here, we revisit the use of the Xpert assay as a marker of concurrent treatment response, as well as treatment success versus treatment failure at the end of 24 weeks therapy.

## Material and Methods

### Human subjects approvals

The study was approved by the Institutional review board of Rutgers University (0120100144), and the Stellenbosch University (N10/01/013). All patients provided written informed consent.

### Patient enrollment, sample collection and processing

We enrolled HIV-negative, smear or Xpert positive, adult pulmonary TB patients at Stellenbosch University as part of the National TB Program in Cape Town, South Africa. All TB cases were treated for a two-month intensive phase with daily fixed-dose combination tablets (Rifafour) containing isoniazid (INH), RIF, ethambutol (EMB) and pyrazinamide (PZA) followed by a four-month continuation phase of daily INH and RIF. Patients with a history of drug susceptible TB or treatment failure were treated with Rifafour plus streptomycin for the first 2 months followed by Rifafour only in month three, and then five months of INH and RIF. Treatment regimens were changed based on drug susceptibility test (DST) results in resistant patients. Each patient was followed for minimum 24 weeks and early morning sputum specimens were collected before (day 0), and during treatment (day 2, week 1, 4, 8, 12, and 24). Approximately one mL of each sputum sample from each time point was decontaminated and concentrated using the NALC-NaOH method as described previously **[19]** [Figure A in S1 File]. The exact quantity of sputum processed, and the volume of the re-suspended pellet varied somewhat according to standard laboratory practice in the first 19 subjects and these 19 samples were excluded from the culture “time to positive analysis”. Thereafter, all pellets were re-suspended in exactly 1mL phosphate buffer, of which 0·5mL was inoculated into mycobacterial growth indicator tubes (MGIT), and the other 0·5mLaliquot was tested by Xpert. After a pre-pilot study phase, the week 12-time point was dropped for Xpert testing but not for culture, leaving insufficient Xpert data to analyze at this point.

We also analyzed sputum samples of 73 patients who were available for a formal follow-up study visit 1 year after the end of treatment. A patient’s clinical outcome was classified as cured if they converted to and maintained sputum culture negativity by week 24 and remained culture negative for 1 year; failed if they were culture positive at week 24 and un-evaluable if contamination caused uncertainty in outcome.

### Study Definitions

“Time to positive” (TTP) was defined as the number of days from time of inoculation into MGIT vial to the first positive culture result. All contaminated cultures were excluded from TTP analysis. As per WHO guidelines, “Time to Culture Negativity” was defined as the number of days from treatment initiation date to the date of the first of two consecutive negative sputum cultures collected at least 30 days apart, **[20]** and further categorized as; TB negativity by week 4; 8; 12; and 24 or TB positive at Week 24. A panel of three investigators who were blinded to the Xpert results determined treatment outcomes. The panel classified each case as follows: definite treatment success (two or more consecutive negative culture results, including a negative week 24 culture, with no intervening positive cultures), probable treatment success (two or more consecutive negative culture results in a subject who lacked a week 24 result due to culture contamination with no intervening positive cultures), Possible treatment success (a single negative culture result at week 24), treatment failure (culture positive at week 24), or un-evaluable (multiple contaminated cultures including week 24). Possible cures are excluded in analyses that consider treatment outcomes.

### Xpert test result definitions

Cycle Threshold (Ct). The number of cycles required for the fluorescent signal from any one of the 5 rpoB (i.e. probe A to E) probes of the Xpert assay to cross a pre-defined fluorescence intensity. The Ct of the first positive probe was used to identify the minimum Ct value of each assay. This definition of minimum Ct value was used (as opposed to a value derived by averaging the Ct values of all five rpoB probes) because it was less likely to be artificially delayed by the presence of Rifampicin resistance, and is consistent with our prior work.**[11, 13]** When TB was not detected by Xpert a CT value of 40 (Ct value above the highest Ct value used to identify TB in the Xpert assay) was assigned for data analysis.

Delta Ct. The minimum rpoB probe Ct value generated by the Xpert assay at a time point of interest minus the minimum rpoB probe Ct value at baseline. When the Xpert Ct value was 40 at the baseline and other subsequent time points, we used an imputed value (instead of zero) of the maximum delta (across all subjects) at that subsequent time point as shown in Table A in S1 File.

Percent closing of baseline deficit (percent closing). This method measured the degree to which an Xpert test at a time point of interest moved towards a negative result compared to the Xpert test at baseline. The percent closing was calculated using the formula: [(Xpert Ct at given time point-baseline Xpert Ct) / (40-baseline Xpert Ct)] x 100. For example, for a baseline Ct = 25 and a week 8 Ct = 35, the percent closing would be 67%, indicating that 67% of the gap between 25 & 40 had been closed at Week 8. When the Xpert Ct value was 40 at the baseline and other subsequent time points, the percent closing value was set to 100% (Table A in S1 File).

### Statistical Analysis

Patient data were analyzed using statistical software Stata (v12), SAS (v9·4) and R (v3·0·1). Baseline differences in Xpert Ct and MGIT TTP by time to negativity category (i.e., ≤ 4 Weeks, ≤8 Weeks, or ≤ 24 weeks) were evaluated using the Kruskal-Wallis test. The association between baseline Ct value and the time to TB negativity was analyzed using a Cox proportional hazard analysis and interval censoring methods. Receiver operating characteristic (ROC) curves were used to plot the diagnostic accuracy of Xpert Ct values at different time points relative to concurrent culture status and to adjudicated treatment outcome. Tests of the non-parametric area under the ROC curve (AUC) compared the performance of the different Xpert parameters using the Mann-Whitney-U-statistic. Multivariate regression was used to create a risk score that combines baseline and post-baseline Xpert Ct measurements [21].

## Results

**Fig 1:**
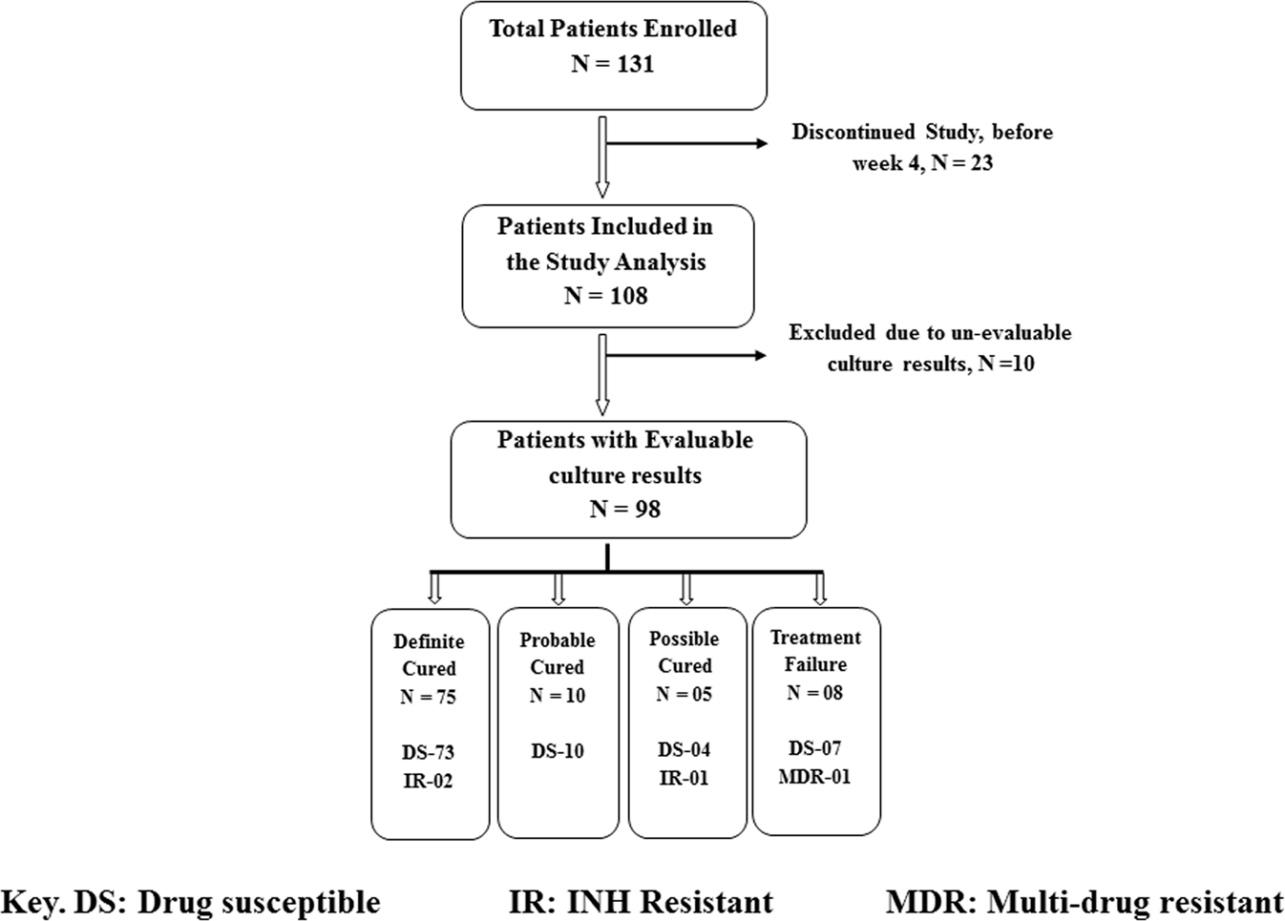
Overview of patient enrollment and adjudicated treatment outcomes at 24 weeks. DS: drug susceptible; IR: INH resistant, MDR: multi drug-resistant.

One hundred thirty-one HIV-negative smear or Xpert positive pulmonary TB patients were enrolled (Fig 1). Twenty-three subjects discontinued very early in the study, and were excluded from analysis. Ten of the 108 subjects were further excluded from the “time to culture negativity” analysis due to un-evaluable culture results. Four of the remaining 98 patients (4.1%) were drug-resistant, and 63 (70·4%) were diagnosed with TB for the first time. Two more patients were excluded due to co-morbidities with other diseases, and the final analyses were conducted with the remaining 96 subjects. For the analysis of adjudicated treatment outcomes, possible treatment success patients were excluded due to their uncertain TB outcome status, and a “treatment success” group consisting of definite and probable treatment success patients was compared against treatment failures. Defaulters were defined as subjects who missed ≥ 30 treatment doses. Based on this definition, there were seven defaulters in the treatment success group and four defaulters in the failed group.

**Fig 2:**
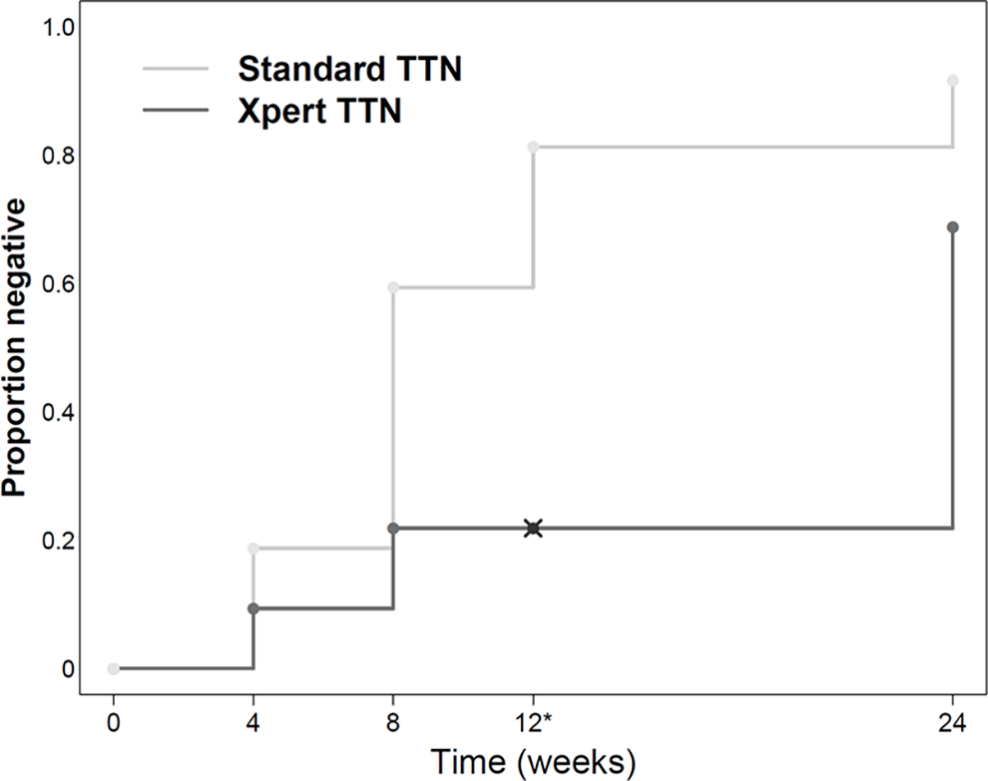
Time to negative results Xpert MTB/RIF Ct relative to MGIT culture. Kaplan-Meyer curves are shown for the time to TB negativity as indicated by two successive negative MGIT cultures versus TB negativity as indicated by a single negative Xpert test. * Xpert testing was not performed at the week 12 time point.

Our overall goal was to identify markers of response to therapy, and treatment success. We compared the time required for conversion of culture to negative to the time required for conversion of the Xpert assay to negative. Kaplan-Meier curves showed that Xpert negativity lagged behind culture negativity (Fig 2), although the lack of Xpert testing at week 12 may have exaggerated this phenomenon between weeks 12, and 24. Eighty-eight of our 96 subjects (91·7%) achieved culture negativity by week 24 [Table B in S1 File] compared to only 66/95 (69.5%) subjects who became Xpert negative over the same period [Table C in S1 File]. We also examined the sputum of 73 subjects who were available for testing one year after the end of their treatment (Table D in S1 File). Xpert produced an error for 2 subjects, leaving 71 with a valid Xpert result. Sixty-four of these 71, had been classified as definite treatment success, 4 as possible treatment success, and 3 as treatment failures at week 24. Eight (12.5%) of 64 patients classified as definite treatment success were Xpert positive one year later. Only one of these positives likely represented relapse or reinfection because only this subject had cultures that were also positive at one year after treatment. All of the possible treatment success subjects were negative by Xpert at the one year time point. Of the three 24 week treatment failures, one was culture and Xpert positive, one was only Xpert positive and one was negative by Xpert and culture at the one year post treatment time point.

**Fig 3:**
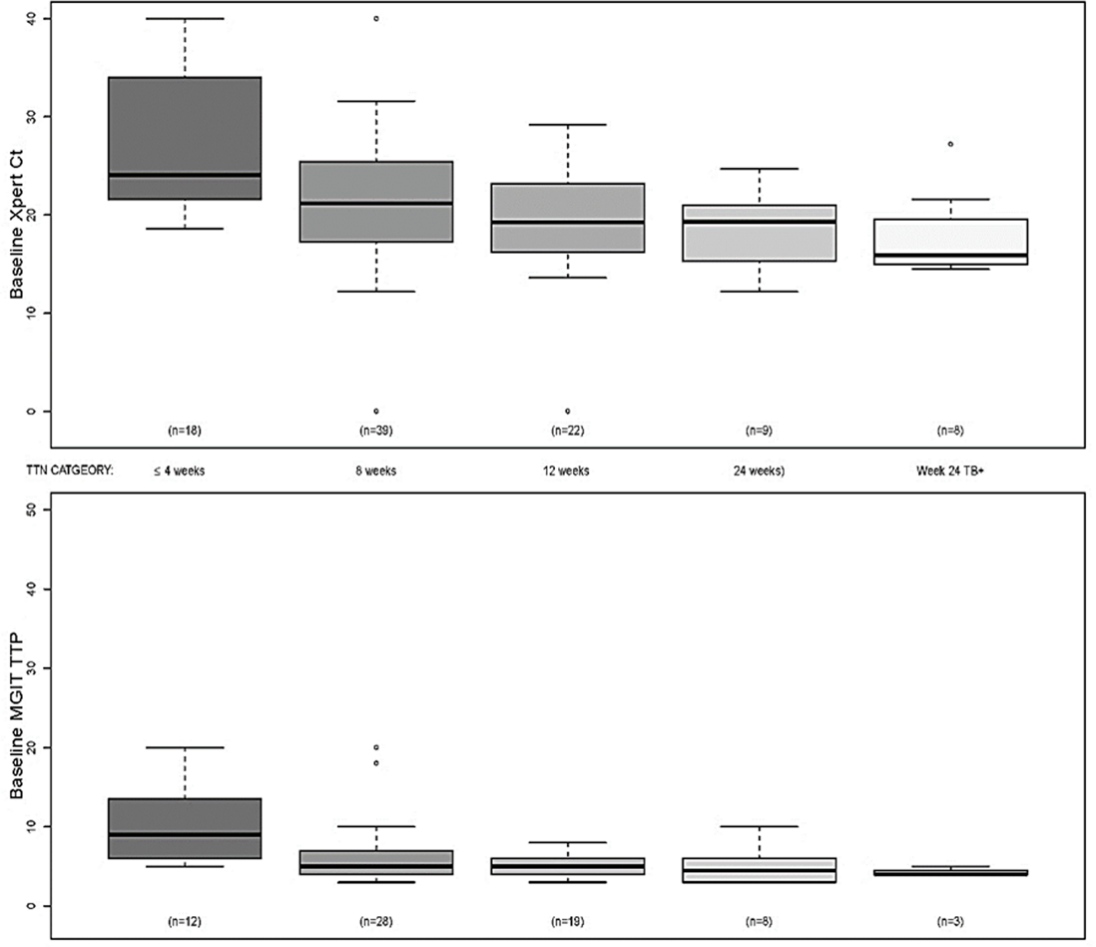
Baseline Xpert and MGIT results and time to culture negativity. The mean, upper and lower mid quartiles, standard deviations, and outliers of the baseline Xpert Ct (top) and baseline MGIT TTP (bottom) are shown for subjects who converted their culture at the indicated time points. All culture negative samples reported as negative at day 42 by MGIT instrument were assigned a TTP value of 45 and all negative Xpert assays were assigned a Ct value of 40. It should be noted that approximately one third of MGIT TTP findings were excluded from the analysis either due to culture contamination or sputum aliquot inconsistencies.

Culture and Xpert both generate quantitative data that can potentially reflect sputum bacterial load. Quantitation is performed by measuring the TTP **[22, 23]** using MGIT culture and by measuring the Ct value using Xpert **[13]**. Baseline Xpert Ct values and baseline MGIT TTP, over time are shown in Fig 3 stratified by time to negativity category. Baseline Xpert Ct values and MGIT TTP were both associated with time to TB culture negativity (p = 0.001 & p = 0.004, respectively). For both tests, patients in the group who became TB negative the soonest had higher values relative to other strata at baseline through day 7, after which the increasing number of culture negative patients made it difficult to distinguish among the strata by means of the TTP. For Xpert Ct, this difference continued past week 4, and the rank ordering of strata was much more pronounced. It should be noted that both MGIT TTP and Xpert Ct are inversely related to bacterial and genome load, respectively [24]. A proportional hazard analysis was performed to calculate the hazard ratio for time-to-negativity as a function of baseline Xpert Ct value (Table E in S1 File). The hazard ratio was 1.06 (p = 0.0023; 95% CI: 1.02,1.09), indicating that the likelihood of earlier conversion to culture negativity improved by 6% with each unit increase in baseline Ct value.

These study results suggest that baseline bacterial load measured by Xpert (base line Ct) might be a useful predictor of later treatment response (Table 1). Baseline Xpert Ct predicted culture negativity at week 4 with 88.2% sensitivity, 51.4% specificity, at week 8, 78.9% sensitivity, 78.9% specificity and at week 24, 75.3% sensitivity, 62.5% specificity. However, a single Xpert Ct result cannot capture the change in bacterial load that occurs with treatment. Therefore, to improve the ability of Xpert to predict culture negativity at critical time points, we further examined two measures of treatment effect: “delta Ct”, which was the difference between the Ct at a time point of interest, and the Ct at day 0; and the percent closing of baseline deficit” (percent closing), which was the percent that the Ct at day 0 moved to the Ct of a negative test (Ct = 40) at the time of interest. This second measure has the potential advantage of not penalizing subjects with high baseline Ct values. However, the ROC curves suggest that—using delta Ct or percent closing measures did not perform appreciably better then baseline Ct at predicting conversion to a negative culture by week 8 (Figure B in S1 File, Table F in S1 File).

**Table 1:** Baseline Xpert Ct value to predict time to negativity status at different time points.

	TTN category	
Baseline values only to predict TTN at week	Sensitivity (TB negative)	Specificity (TB positive)
4	88.2% (15/17)	51.4%(38/74)
8	78.9% (31/53)	78.9% (30/38)
24	75.9% (63/83)	62.5% (5/8)

**Fig 4:**
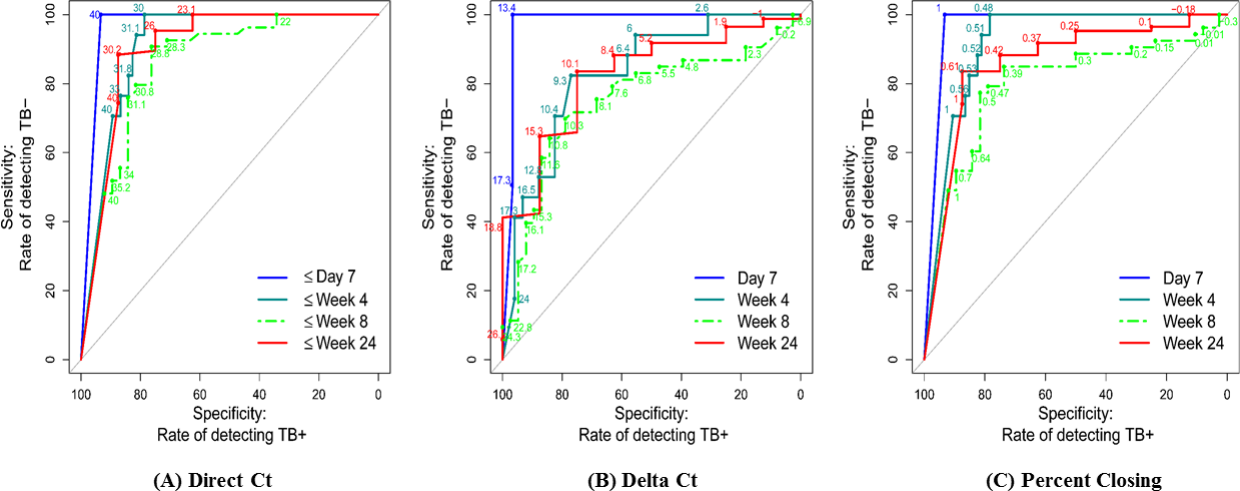
ROC curve for direct Xpert CT, delta CT and percent closing relative to culture negativity at the same time point. Assessment of direct Xpert Ct (A), delta Ct (B) and percent closing (C) values to predict the same-day culture negativity at day 7, week 4, week 8, and week 24. ROC curve results for direct Xpert Ct (optimal cut off approximately ≥30) at Day 7, AUC = 96.7 (95% CI 94.1, 99.3); at week 4, AUC = 91.2 (95% 85.8, 96.6); and week 8 AUC = 86.0 (95% 77.9, 94.2) and at week 24, AUC = 90.2 (95% 75.0, 100.0). ROC curve results for the delta Ct (optimal cut off values of approximately ≥ 10) at day 7, AUC = 97.5 (95% CI 94.5, 100.0); at week 4, AUC = 82.8 (95% 72.5, 93.1); at week 8, AUC = 75.2 (95%CI 64.8, 85.6); and at week 24, AUC = 82.2 (95%CI 67.9, 96.5). ROC curve results for the percent closing of the baseline Ct deficit at day 7, AUC = 96.6 (95% CI 94.0, 99.2); week 4, AUC = 91.6 (95%CI 86.2, 96.9); week 8, AUC = 79.8(70.3, 89.4); and at week 24, AUC = 86.0 (95%CI 71.3, 100.0).

We also examined the ability of Xpert as a marker of whether contemporaneous cultures (cultures collected on the same day the Xpert test was performed) would ultimately prove to be negative. This measure is potentially quite useful given the several week delay between culture collection and a definitive negative culture test. This analysis showed that the Xpert Ct measured on the same day that a culture was obtained (direct Ct) was strongly associated with current culture status, with ROC curve AUCs at day 7 of 96.7 (95% CI 94.1, 99.3), at week 4 of 91.2 (95% CI 85.8, 96.6), at week 8 of 86.0 (95% CI 77.9, 94.2) and at week 24 of 90.2 (95% CI 75.0, 100.0) [Fig 4(A), Table Ga in S1 File]. The optimal point, measured in terms of maximizing the sum of sensitivity and specificity, for a Ct cutoff rule confirming culture negativity was approximately ≥30 at the three later time points. ROC curves that used delta Ct (optimal cutoff ≥10) relative to current status (negative/positive) indicated a somewhat lower accuracy, with a ROC AUC of 75.2 (95% CI 64.8, 85.6) at week 8 and a ROC curve AUC of 82.2 (95% CI 67.9, 96.5) at week 24 [Fig 4(B); Table Gb in S1 File]. For the percent closing measure, the ROC curve AUCs were somewhat closer to the performance of direct Ct, with a ROC curve AUC of 79.8 (95% CI 70.3, 89.4) at week 8, and a ROC curve AUC 86 (95% CI 71.3, 100.0) at week 24 [Fig 4(C); Table Gc in S1 File].

**Fig 5:**
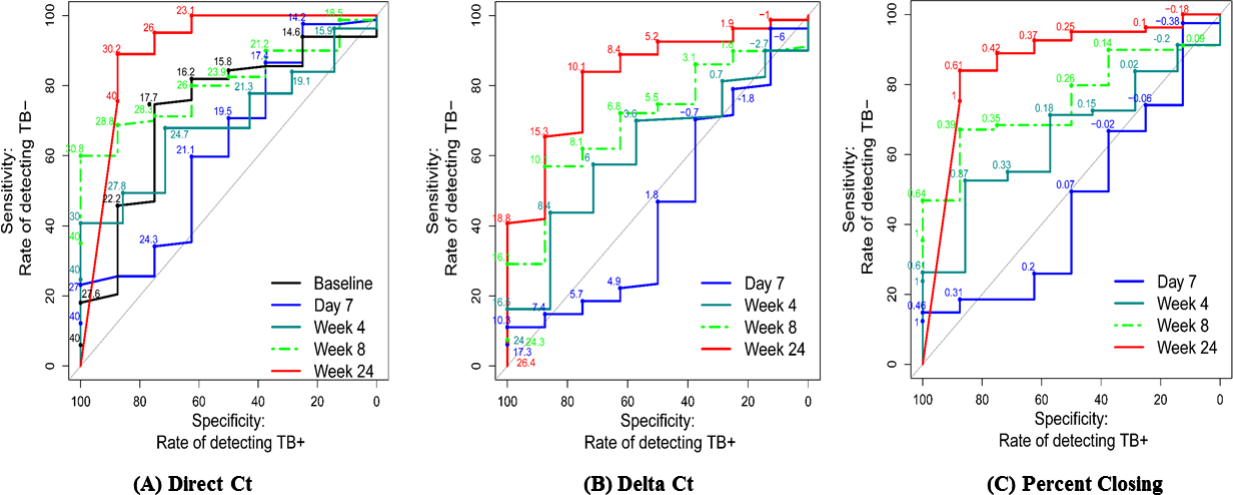
ROC curve for baseline Ct, direct Ct, delta Ct and percent closing deficit to predict treatment failure at the end of the treatment. Evaluation of ROC curves for direct Xpert Ct showed AUC = 80.2 (95%CI 69.2, 91.3) at week 8, and AUC = 90.2 (95%CI 75.2, 100.0) at week 24. ROC curves for delta Ct demonstrated AUC = 70.2 (95%CI 54.1, 86.2) at week 8 and AUC = 82.6 (95% CI 68.1, 97.0) at week 24. ROC curves for the percent closing deficits at week 8, AUC = 75.2 (95%CI 62.2, 88.1) and at week 24, AUC = 86.2 (95%CI 71.6, 100.0).

Week 24 culture negativity is currently the best available predictive marker for treatment success **[25]**. One of the potentially best uses of a sputum biomarker would be to predict which TB patients will have treatment success after 24 weeks of treatment and which will fail or require more treatment. Such a biomarker would be most useful if it could be measured relatively early into therapy when treatment regimens could be modified, and it would also be useful at the end of treatment to identify patients who need more prolonged therapy. We studied whether a single Ct value could be used in this manner either at baseline (baseline Ct) and during treatment (direct Ct). The ROC curves suggested that the baseline Ct value as well as the direct Ct values were not predictive of treatment success (vs. failure) until week 8, AUC = 80.2 (95% CI 69.2, 91.3), and they were an even better predictor at week 24, AUC = 90.2 (95% CI 75.2, 100.0) [Fig 5A, Table Ha in S1 File], with a threshold of 30 providing a sensitivity of 89% and a specificity of 75%. Using either the delta Ct, or percent closing measures did not drastically improve the ROC curves in this analysis (Fig 5(B) and 5(C); Table Hb and Hc in S1 File).

**Fig 6:**
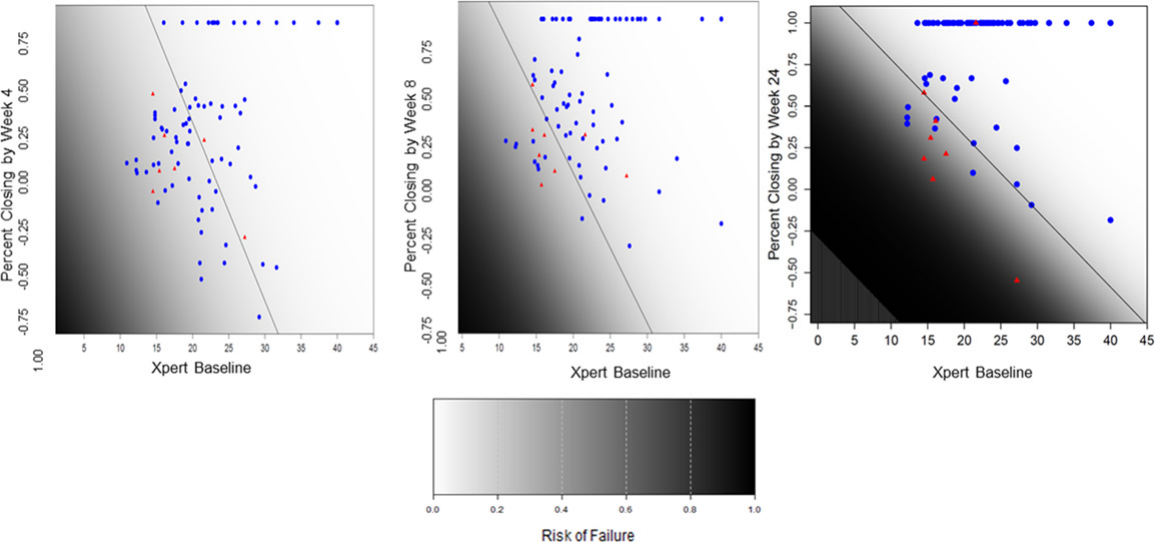
Combined measures of Ct plus percent closing to predict treatment success versus treatment failure. This figure represents a model that include only two covariates: baseline Xpert Ct and percent closing at 4, 8 and 24 weeks (for panels a, b and c respectively). Optimal thresholds are then defined using Youden’s index (on the risk scores provided from the logistic regression models), with sensitivity and specificity estimates derived from application of leave-on-out cross-validation. The Xpert Ct and percent closing measure for each cured case (blue dots) and each treatment failure case (red dots) are shown. The optimal thresholds to predict treatment success and treatment failure using both Xpert Ct and percent closing at each time point are indicated by a diagonal black line. Color gradation from black to white indicate increase chances of culture negativity. Cross validated results using combination of a direct Xpert Ct and percent closing using Youden’s index to predict treatment failure showed 71.4% sensitivity and 42.5% specificity at week 4, 62.5% sensitivity and 84.0% specificity at week 8, and 75% sensitivity 88.9% specificity at week 24.

Since neither delta Ct nor the percent closing of baseline deficit performed better than direct Ct as a predictor of culture negativity at the same day of treatment (Fig 5) or at the end of therapy, we considered the possibility that these treatment effect measures would be better predictors of culture outcomes if used in combination with a baseline Xpert Ct measurement. Therefore, we examined optimal thresholds to predict concurrent culture negativity and treatment failure using combined parameters. In some cases, these composite rules made it possible to predict each outcome with somewhat better sensitivity and specificity. As shown in Table 2, cross validated results using combinations of baseline Ct and either delta Ct or percent closing with Youden’s index only appeared equivalent to direct Ct alone for tests of concurrent culture negativity or treatment failure. However, the combined parameters did modestly improve the sensitivity and specificity for predicting treatment failure compared to baseline Ct alone, and in some cases also modestly improved the sensitivity and specificity compared to direct Ct, although sensitivity compared to direct Ct was also adversely affected in some cases (Table 2 and Fig 6). For example, cross- validated results using only baseline Ct as a predictor of treatment failure had a relatively poor sensitivity of 62.5% and a specificity of 75.3% (Table 2), while using a combination of baseline Ct and percent closing measured at week 24 gave a sensitivity for treatment failure of 75% and a specificity of 88.9%. We also analyzed multiple variable logistic regression models (Table I in S1 File) using week 4, week 8 and week 24 data. Models including these variables demonstrated that delta Ct or percent closing were important predictors of treatment outcome (at each time point) after adjustment for baseline direct Ct.

**Table 2:** An optimal threshold to forecast culture negativity at the same visit and treatment failure using Xpert Ct at the end of the treatment.

		Concurrent culture negativity			Adjudicated treatment failure by week 24		
		Culture Negative(Sensitivity)	Culture Positive(Specificity)	Selectedcutpoint	TreatmentFailure(Sensitivity)	TreatmentSuccess(Specificity)	Selectedcutpoint
**XpertCt at given time**	**Baseline**	NA	NA	NA	62.5% (5/8)	75.3% (61/81)	17.6
	**Week 4**	94.1% (16/17)	79.7% (59/74)	29.8	71.4% (5/7)	40.0% (32/80)	29.7
	**Week 8**	88.6% (47/53)	76.3% (29/38)	28.6	87.5% (7/8)	59.0% (47/79)	30.6
	**Week 24**	87.9% (73/83)	75% (6/8)	29.7	75.0% (6/8)	88.9% (72/81)	29.7
**Xpert baseline Ct & Delta Ct**	**Week 4**	94.1% (16/17)	79.7% (59/74)	NA	57.1% (4/7)	56.3% (54/80)	NA
	**Week 8**	88.6% (47/53)	73.6% (28/38)	NA	75.0% (6/8)	67.1% (53/79)	NA
	**Week 24**	89.2% (74/83)	75.0% (6/8)	NA	75.0% (6/8)	88.9% (72/81)	NA
**Xpert baseline Ct and percent closing**	**Week 4**	94.1% (16/17)	81.1% (60/74)	NA	71.4% (5/7)	46.3% (37/80)	NA
	**Week 8**	83.0% (44/53)	71% (26/38)	NA	62.5% (5/8)	84.8% (67/79)	NA
	**Week 24**	89.2% (74/83)	75.0% (6/8)	NA	75.0% (6/8)	88.9% (72/81)	NA

## Discussion

Our study demonstrates that quantitative data produced by the Xpert assay may have utility for monitoring TB treatment. For example, a quantitative Xpert assay result could aide in the determination of treatment success at or near the end of treatment. In this regard, the assay’s two hour time to result might be particularly useful, as patients would not need to wait for culture results and risk being lost to follow-up. While conditions that reflect high bacterial load such as AFB smear positivity, and extensive or cavitary TB are recognized as poor prognostic factors for TB treatment, **[1, 5]** our study demonstrates for the first time the strong relationship between initial bacterial load measured by a baseline Xpert Ct and subsequent time to culture conversion. The quantitative information from even a single test performed before starting treatment proved to be a relatively good predictor of time to culture conversion. Individual tests performed between 4 and 24 weeks of treatment were also good at predicting concurrent (same day) culture negativity and patients who would ultimately be adjudicated as treatment failures. These predictions could be performed even though a relatively large proportion (30.6%) of study subjects had detectable *M*. *tuberculosis* DNA at the end of treatment. Adding either delta Ct or percent closing to the baseline Ct produced a combined measure that moderately improved the predictive ability treatment failure over baseline Ct alone. However, the observed improvement was relatively modest and our small sample size precluded a useful statistical evaluation of the added benefit of these combined measures over baseline Ct.

Prior studies have also noted that patients can remain Xpert positive at the end of treatment. **[15, 16].** However, these studies were unable to validate Xpert as a useful treatment biomarker, possibly because they appear to have not performed quantitative comparisons between Xpert and same day culture status, or because (we speculate) there was less uniformity in the way sputum samples were collected and processed for Xpert testing. Additionally, prior studies did not report treatment outcome information, nor examine combined measures of direct Ct with delta Ct or percent closing. To our knowledge, our study is the first to demonstrate the potential utility of the Xpert assay in a treatment monitoring capacity.

This study has several limitations. First, it was a relatively small exploratory study performed at a single site, with a small number of treatment failures with varying levels of treatment compliance. It included only four drug resistant cases. Thus, our results will need to be confirmed by a larger study. Second, the 16% culture contamination rate in our study was higher than the usually reported contamination rate of 2% to 5%, as a result, contaminated cultures were evaluated as TB positive or negative by Capilia testing but they had to be excluded from analyses that utilized TTP values. It was unfortunate that our study did not include a week 16 time point. There is considerable interest in developing therapeutic regimens that can provide a reliable cure after only 16 weeks of therapy. Future studies should evaluate whether the approach outlined here is useful at 16 weeks to stratify patients by low and high risk for treatment failure, which may be useful to explore treatment-shortening strategies. Finally, we were only able to identify 73 subjects to examine Xpert Ct positivity at the one-year post-treatment time point as the rest were lost to follow up after treatment completed (this last time point was not anticipated in the initial study design).

It was surprising that the results of a single Xpert assay at a single time point performed at least as well as a measurement of delta Ct, and percent closing at predicting culture conversion, and treatment outcome at most time points. The delta Ct, and percent closing measures were expected to provide an indication of treatment effect, since appropriate TB treatment should reduce the *M*. *tuberculosis* bacterial load, and eventually the *M*. *tuberculosis* DNA load present in sputum. We expected that these measures of treatment efficacy would be a major component of any predictor of culture conversion or treatment outcomes. Indeed like previous studies, **[15, 16]** we did demonstrate a fall in *M*. *tuberculosis* DNA with treatment. We believe that the additional predictive benefit of delta Ct and percent closing was relatively small for several reasons. First, our study included very few subjects with drug resistant TB. We would expect that treatment measures might be most important for subjects with drug resistance, because drug resistant TB is likely be at increased risk for treatment failure even in “good prognosis” “high Ct” TB patients. Second, we only had 12 treatment defaulters in our study. Delta Ct or percent closing might be expected to be particularly useful at detecting subjects who were not adhering to therapy; and therefore, would be unlikely to show a good treatment response. Third, the importance of pre-treatment bacterial load on time to culture conversion and ultimate treatment success versus treatment failure, as shown in Table 1, appears to have been underestimated by us and others. **[2]**

Our study confirms that the initial bacterial load is a critical predictor of treatment outcomes as reported previously. It also appears that the relationship between initial bacterial load and subsequent time to culture conversion and treatment outcome extends along the entire dynamic range of possible bacterial loads. In conclusion, the Xpert assay shows considerable promise as a biomarker for response to TB treatment. Further larger studies will be needed to validate this work and to more precisely define the assay Ct and percent closing parameters that provide the best sensitivity and specificity for its predictions. If Ct’s refined in a larger study provide adequate sensitivity and specificity as well as adequate predictive values in appropriate target populations, then Xpert Ct could be a useful tool for clinicians evaluating an individual patient’s response to TB treatment, as it would allow treatment regimens to be modified based on same-day information. It also has the potential to make possible a wider range of clinical trial design strategies, particularly allowing dose-ranging or early bactericidal activity studies to use adaptive treatment allocation designs such as those commonly used in cancer research.

## Supporting Information

S1 File **Figure A in S1 file: Flow chart for specimen collection and processing for MGIT culture and the Xpert assay.** A deeply expectorated early morning sputum specimen was collected at each time point before and during the anti-TB therapy and processed by standard NaLC-NaOH method for MGIT and Xpert analysis. **Table A in S1 file. Imputed values for delta Ct and percent closing decificit when Xpert was negative Note:** 1. Green cells in the following table contain imputed values. 2. When TB was not detected by Xpert a CT value of 40 (Ct value above the highest Ct value used to identify TB in the Xpert assay) was imputed for statistical analysis. **Table B in S1 file. Time to TB Negativity by MGIT culture (N = 108). Table C in S1 file. Xpert MTB/RIF results of culture evaluable subjects (N = 108). Table D in S1 file. One year end of the treatment follow up, Xpert MTB/RIF assay results. Table E in S1 file. Hazard ratio using baseline Xpert Ct as a predictor of time-to-culture negativity demonstrated by Cox proportional hazard analysis. Figure B in S1 file. ROC curve of direct Xpert Ct (A), delta Ct (B) and percent closing (C) as a predictor of culture conversion by week 8.** Evaluation of direct Xpert Ct at baseline, day 7, week 4, and week 8 (A), and delta Ct (B) and percent closing (C) at day 7, week 4, and week 8 to predict culture conversion by week 8. ROC curve results for direct Xpert Ct AUC = 68.8 (95% CI 58.1, 79.5), Xpert Ct at Day 7, AUC = 70.3 (95% CI 59.7, 80.9); week 4, AUC = 74.9 (95% CI 64.9, 84.9) and week 8, AUC = 86.0 (95% CI 77.9, 94.2). ROC curves for Delta Ct at Day 7, AUC 61.3(95% CI 49.4, 73.2); week 4, AUC = 67.9 (95% CI 56.7, 79.1) and week 8, AUC = 75.2 (95% CI 64.8, 85.6). ROC curves for percent closing deficit at Day 7, AUC = 62.1 (95% CI 50.5, 73.8); week 4, AUC = 71.3 (95% CI 60.6, 81.9) and week 8, AUC = 79.8 (95% CI 70.3, 89.4). **Table F in S1 file. Area Under the curve (AUC) for Xpert Ct (Fa), delta Ct (Fb) and percent closing Ct (Fc) using culture negativity status at Week 8 as outcome. Note:** Culture negative status was defined based on TTN category. Rules for defining negative status for each parameter are listed in these tables. **Table G in S1 file. AUC values for Xpert direct Ct (Ga), delta Ct (Gb) and percent closing Ct (Gc) ROC curves using culture negativity status at the same visit. Note:** Culture negative status was defined based on TTN category. Rules for defining negative status for each parameter are listed in these tables. **Table H in S1 file. Area Under the curve (AUC) values for Xpert direct Ct (Ha), delta Ct (Hb) and percent closing Ct (Hc) ROC curves using treatment failure status at the end of the therapy. Table I in S1 file. Multiple variable logistic regression models.**
(DOCX)
