# Supplementary material for: Bacterial Loads Measured by the Xpert MTB/RIF Assay as Markers of Culture Conversion and Bacteriological Cure in Pulmonary TB
Source: PLoS One. 2016 Aug 10;11(8):e0160062. doi: 10.1371/journal.pone.0160062 (PMC4980126; doi:10.1371/journal.pone.0160062)
Supplement: S1 File — Figure A in S1 file: Flow chart for specimen collection and processing for MGIT culture and the Xpert assay. A deeply expectorated early morning sputum specimen was collected at each time point before and during the anti-TB therapy and processed by standard NaLC-NaOH method for MGIT and Xpert analysis. Table A in S1 file. Imputed values for delta Ct and percent closing decificit when Xpert was negative Note: 1. Green cells in the following table contain imputed values. 2. When TB was not detected by Xpert a CT value of 40 (Ct value above the highest Ct value used to identify TB in the Xpert assay) was imputed for statistical analysis. Table B in S1 file. Time to TB Negativity by MGIT culture (N = 108). Table C in S1 file. Xpert MTB/RIF results of culture evaluable subjects (N = 108). Table D in S1 file. One year end of the treatment follow up, Xpert MTB/RIF assay results. Table E in S1 file. Hazard ratio using baseline Xpert Ct as a predictor of time-to-culture negativity demonstrated by Cox proportional hazard analysis. Figure B in S1 file. ROC curve of direct Xpert Ct (A), delta Ct (B) and percent closing (C) as a predictor of culture conversion by week 8. Evaluation of direct Xpert Ct at baseline, day 7, week 4, and week 8 (A), and delta Ct (B) and percent closing (C) at day 7, week 4, and week 8 to predict culture conversion by week 8. ROC curve results for direct Xpert Ct AUC = 68.8 (95% CI 58.1, 79.5), Xpert Ct at Day 7, AUC = 70.3 (95% CI 59.7, 80.9); week 4, AUC = 74.9 (95% CI 64.9, 84.9) and week 8, AUC = 86.0 (95% CI 77.9, 94.2). ROC curves for Delta Ct at Day 7, AUC 61.3(95% CI 49.4, 73.2); week 4, AUC = 67.9 (95% CI 56.7, 79.1) and week 8, AUC = 75.2 (95% CI 64.8, 85.6). ROC curves for percent closing deficit at Day 7, AUC = 62.1 (95% CI 50.5, 73.8); week 4, AUC = 71.3 (95% CI 60.6, 81.9) and week 8, AUC = 79.8 (95% CI 70.3, 89.4). Table F in S1 file. Area Under the curve (AUC) for Xpert Ct (Fa), delta Ct (Fb) and percent closing Ct (Fc) using culture ne [file pone.0160062.s001.docx]

**Supplementary Materials**

**Supplementary Methods.**

**Positive, TTP noted**

**AFB Smear or Xpert Positive Pulmonary TB Patients Subjects**

**Day 0**

**Day 2**

**Day 7**

**Day 28**

**Day 56**

**Day 84**

**Day168**

**Early morning deeply expectorated sputum specimens (N=108)**

**1 ml sputum processed to pellet (std. NaLC-NaOH treatment)**

**Liquid Culture (MGIT) Inoculated immediately**

**Pellet frozen at -80 transported to RBHS on dry ice**

**Negative**

**Xpert MTB/RIF**

**Confirmation with in-house**

**PCR &/or Capilia**

**In case of contamination TTP excluded from the final analysis**

**0.5 ml**

**0.5 ml**

**Figure A.** **Flow chart for specimen collection and processing for MGIT culture and the Xpert assay**

A deeply expectorated early morning sputum specimen was collected at each time point before and during the anti-TB therapy and processed by standard NaLC-NaOH method for MGIT and Xpert analysis.

| **SUBJECT ID** | **Xpert_base** | **Xpert_Day_7** | **Xpert_Week_4** | **Xpert_Week_8** | **Xpert_Week_24** | **Delta_Day_7** | **Delta_Week_4** | **Delta_Week_8** | **Delta_Week_24** | **Pt_closing_Day_7** | **Pt_closing_Week_4** | **Pt_closing_Week_8** | **Pt_closing_Week_24** |
| --- | --- | --- | --- | --- | --- | --- | --- | --- | --- | --- | --- | --- | --- |
| 41 | 40 | 40 | 40 | 40 | 40 | 17.3 | 24 | 24.3 | 26.4 | 1 | 1 | 1 | 1 |
| 93 | 40 | 40 | 40 | 40 | 40 | 17.3 | 24 | 24.3 | 26.4 | 1 | 1 | 1 | 1 |
| 131 | 40 | 40 | 40 | 40 | 40 | 17.3 | 24 | 24.3 | 26.4 | 1 | 1 | 1 | 1 |
| 136 | 40 | 32.1 | 40 | 33.1 | 40 | -7.9 | 24 | -6.9 | 26.4 | -0.198 | 1 | -0.173 | 1 |
| 150 | 40 | 40 | 40 | 40 | 32.7 | 17.3 | 24 | 24.3 | -7.3 | 1 | 1 | 1 | -0.183 |

**Table A.** **Imputed values for delta Ct and percent closing decificit when Xpert was negative**

**Note:**

1. Green cells in the following table contain imputed values

2. When TB was not detected by Xpert a CT value of 40 (Ct value above the highest Ct value used to identify TB in the Xpert assay) was imputed for statistical analysis

**Supplementary Results**

| **^2^Time to TB culture Negativity** | **^3^Not Cured**  **(N=8)** | **^3^Possible Treatment**  **Success**  **(N=7)** | **^3^Probable**  **Treatment**  **Success**  **(N=10)** | **^3^Definite Treatment**  **Success**  **(N=75)** | **^3^Unevaluable**  **(N=8)** | **All Catagories**  **(N=108)** |
| --- | --- | --- | --- | --- | --- | --- |
| Day 2 | 0 | 0 | 1 (10.0%) | 0 | 0 | 1 (0.9%) |
| Day 7 | 0 | 0 | 0 | 3 (4.0%) | 0 | 3 (2.8%) |
| Week 4 | 0 | 0 | 3 (30.0%) | 11 (14.7%) | 0 | 14 (13.0%) |
| Week 8 | 0 | 0 | 4 (40.0%) | 35 (46.7%) | 0 | 39 (36.1%) |
| Week 12 | 0 | 0 | 2 (20.0%) | 20 (26.7%) | 0 | 22 (20.4%) |
| Week 24 | 0 | 4 (57.1%) | 0 | 5 (6.7%) | 0 | 9 (8.3%) |
| TB+at week 24 | 8 (100%) | 0 | 0 | 0 | 0 | 8 (7.4%) |
| Unevaluable | 0 | 2 (28.6%) | 0 | 0 | 8 (100%) | 10 (9.3%) |
| Excluded | 0 | 1 (14.3) | 0 | 1 (1.3) | 0 | 2 (1.9%) |

**^1^Table B.** **Time to TB Negativity by MGIT culture (N=108)**

^1^Time to culture negativity results among the 108 patients who completed at least 4 weeks of anti-TB therapy is cross-tabulated against the week 24 adjudicated outcome**.** Among the 96 TB subjects with a time to TB negativity status, 88 (91.7%) achieved TB negativity by week 24. Eight (8.3%) patients were TB positive at the end of 24 weeks

^2^Measured by MGIT

^3^Adjucated week 24 outcome

| **Status**  **Based on TB culture** | **No of Subjects** | **^2^RIF Sensitive** | **RIF Resistant** | **Mean (SD)**  **Ct at**  **Baseline** | **Xpert(-) at**  **Week 24** | **Xpert(+) at**  **Week 24** | **Week 24 Error** | **Week 24 Missing** |
| --- | --- | --- | --- | --- | --- | --- | --- | --- |
| Definite Cure | 74 | 74 | 0 | 20.9 (5.4) | 55/73 ^4^(75.3%) | 18/73(24.7%) | 1 | 0 |
| Probable Cure | 10 | 10 | 0 | 26.4 (7.0) | 8/10 (80.0%) | 2/10 (20.0%) | 0 | 0 |
| Possible Cure | 4 | 3 | 1 | 20.6 (2.8) | 2/4 (50.0%) | 2/4 (50.0%) | 0 | 0 |
| Not Cured | 8 | 7 | 1 | 17.8 (4.4) | 1/8 (12.5%) | 7/8 (87.5%) | 0 | 0 |
| Unevaluable/excluded | 12 | 12 | 0 | 20.6 (4.2) | 4/6 (66.7%) | 2/6 (33.3%) | 0 | 6 |
| Total | 108 | 99 | 2 | 21.9 (6.6) | 70/101  (69.3%) | 31/101  (30.7%) | 1 | 6 |

**^1^Table C. Xpert MTB/RIF results of culture evaluable subjects (N=108)**

^1^Xpert Ct results of 108 patients at the end of therapy are tabulated. The baseline mean Xpert Ct values for definitely, probably, possibly cured and treatment failure (not cured) patients are shown. Compared to cured subjects, 3-4 Ct (> a log) difference with high bacterial load was observed in mean and median Ct values in non-cured patients’ specimens at the baseline. MTB DNA was not detected in 55/73 (75.3%) definitely cured, 8/10 (80%) probably cured and 2/4 (50%) possibly cured patients at the end of the treatment. In contrast, MTB DNA was not detected only in 1/8 (12.5 %) and detected in 7/8 (87.5%) of treatment failure cases at the end week 24.

^2^RIF = Rifampicin

**^3^**Ct = Cycle Threshold

**^4^**Subjects with “error” or missing Xpert result at week 24 were excluded from the percent calculation.

| **One year end of the treatment follow up**  **N = 73/96** | | | | | |
| --- | --- | --- | --- | --- | --- |
| **Definite**  **Treatment Success**  **N = 64/73** | | **Possible Treatment Success**  **N = 4/73** | **Treatment failure**  **N = 3/73** | | **Error/Invalid**  **(Excluded)**  **N = 2/73** |
| **Xpert Negative**  **for TB**  **N = 56**  **(84.4%)** | **Xpert Positive**  **for TB**  **N = 8**  **(12.5%)** | **Xpert Negative**  **for MTB**  **N=4**  **(100%)** | **Xpert Negative for TB**  **N = 1 (33.3%)** | **Xpert Positive for MTB**  **N = 2 (66.7%)** |  |

**Table D. One year end of the treatment follow up, Xpert MTB/RIF assay results**

|  | **Coefficient** | **Hazard Ratio** | **P-value** | **95% CI** |
| --- | --- | --- | --- | --- |
|  |  |  |  |  |
| **Baseline**  **Xpert Ct** | 0.0554 | 1.05698 | 0.0023 | 1.022, 1.094 |

**Table E. Hazard ratio using baseline Xpert Ct as a predictor of time-to-culture negativity demonstrated by Cox proportional hazard analysis.**

Culture conversion by week 8 is commonly used as an early indicator of treatment success including drug susceptibility **(**[**1-3**](#_ENREF_1)**)**. Therefore, ROC curves of baseline direct Xpert Ct, and ROC curves of the direct Xpert Ct, delta Ct and percent closing at day 7, week 4 and week 8 were assessed as predictors of culture conversion by week 8 (S2 Fig).


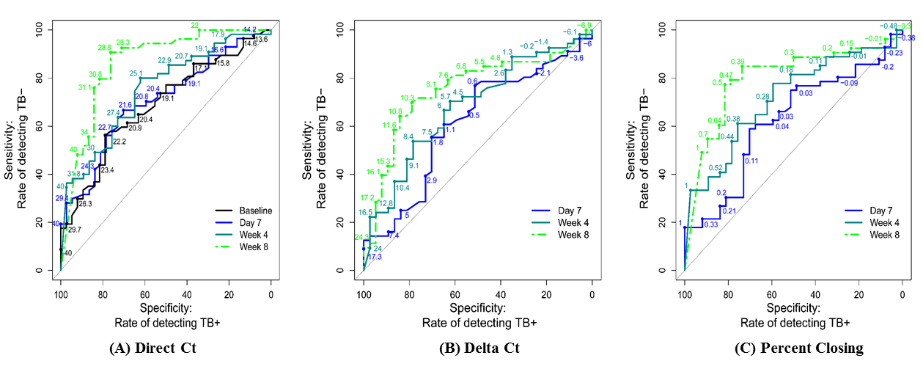


**Figure B.** **ROC curve of direct Xpert Ct (A), delta Ct (B) and percent closing (C) as a predictor of culture conversion by week 8.** Evaluation of direct Xpert Ct at baseline, day 7, week 4, and week 8 (A), and delta Ct (B) and percent closing (C) at day 7, week 4, and week 8 to predict culture conversion by week 8. ROC curve results for direct Xpert Ct AUC=68.8 (95% CI 58.1, 79.5), Xpert Ct at Day 7, AUC=70.3 (95% CI 59.7, 80.9); week 4, AUC=74.9 (95% CI 64.9, 84.9) and week 8, AUC=86.0 (95% CI 77.9, 94.2). ROC curves for Delta Ct at Day 7, AUC 61.3(95% CI 49.4, 73.2); week 4, AUC=67.9 (95% CI 56.7, 79.1) and week 8, AUC=75.2 (95% CI 64.8, 85.6). ROC curves for percent closing deficit at Day 7, AUC=62.1 (95% CI 50.5, 73.8); week 4, AUC=71.3 (95% CI 60.6, 81.9) and week 8, AUC=79.8 (95% CI 70.3, 89.4).

**Table Fa. Xpert Ct**

| **TTN Category** | **AUC** | **Number TB+** |
| --- | --- | --- |
| **Baseline** | 68.8 (58.1,79.5) | 38 |
| **Day 7** | 70.3 (59.7,80.9) | 37 |
| **Week 4** | 74.9 (64.9,84.9) | 37 |
| **Week 8** | 86.0 (77.9,94.2) | 38 |

**Table Fb. Xpert delta Ct**

| **TTN Category** | **AUC** | **Number TB+** |
| --- | --- | --- |
| **Day 7** | 61.3 (49.4,73.2) | 37 |
| **Week 4** | 67.9 (56.7,79.1) | 37 |
| **Week 8** | 75.2 (64.8,85.6) | 38 |

**Table Fc. Xpert “Percent closing Ct”**

| **TTN Category** | **AUC** | **Number TB+** |
| --- | --- | --- |
| **Day 7** | 62.1 (50.5,73.8) | 37 |
| **Week 4** | 71.3 (60.6,81.9) | 37 |
| **Week 8** | 79.8 (70.3,89.4) | 38 |

**Table F. Area Under the curve (AUC) for Xpert Ct (Fa), delta Ct (Fb) and percent closing Ct (Fc) using culture negativity status at Week 8 as outcome**

**Note:** Culture negative status was defined based on TTN category. Rules for defining negative status for each parameter are were listed in these tables.

**Ga Xpert Ct**

| **TTN Category** | **AUC** | **Number TB+** |
| --- | --- | --- |
| **</=Day 7** | 96.7 (94.1,99.3) | 90 |
| **</=Week 4** | 91.2 (85.8,96.6) | 75 |
| **</=Week 8** | 86.0 (77.9,94.2) | 38 |
| **</=Week 24** | 90.2 (75,100) | 8 |

**Gb. Xpert delta Ct**

| **TTN Category** | **AUC** | **Number TB+** |
| --- | --- | --- |
| **</=Day 7** | 97.5 (94.5,100) | 90 |
| **</=Week 4** | 82.8 (72.5,93.1) | 75 |
| **</=Week 8** | 75.2 (64.8,85.6) | 38 |
| **</=Week 24** | 82.2 (67.9,96.5) | 8 |

**Gc. Xpert “Percent closing Ct”**

| **TTN Category** | **AUC** | **Number TB+** |
| --- | --- | --- |
| **</=Day 7** | 96.6 (94,99.2) | 90 |
| **</=Week 4** | 91.6 (86.2,96.9) | 75 |
| **</=Week 8** | 79.8 (70.3,89.4) | 38 |
| **</=Week 24** | 86.0 (71.3,100) | 8 |

**Table G. AUC values for Xpert direct Ct (Ga), delta Ct (Gb) and percent closing Ct (Gc) ROC curves using culture negativity status at the same visit.**

**Note:** Culture negative status was defined based on TTN category. Rules for defining negative status for each parameter are were listed in these tables.

**Ha. Xpert direct Ct**

| **TF Category** | **AUC** | **Number TB+** |
| --- | --- | --- |
| **Baseline** | 72.7 (53.5,91.9) | 8 |
| **Day 7** | 62.2 (39.9,84.5) | 8 |
| **Week 4** | 69.1 (53.1,85.2) | 7 |
| **Week 8** | 80.2 (69.2,91.3) | 8 |
| **Week 24** | 90.2 (75.2,100) | 8 |

**Hb. Xpert delta Ct**

| **TTN Category** | **AUC** | **Number TB+** |
| --- | --- | --- |
| **Day 7** | 45.1 (21.3,69) | 8 |
| **Week 4** | 61.5 (41.6,81.5) | 7 |
| **Week 8** | 70.2 (54.1,86.2) | 8 |
| **Week 24** | 82.6 (68.1,97) | 8 |

**Hc. Xpert “percent closing”**

| **TTN Category** | **AUC** | **Number TB+** |
| --- | --- | --- |
| **Day 7** | 45.7 (23.2,68.2) | 8 |
| **Week 4** | 64.6 (46.7,82.6) | 7 |
| **Week 8** | 75.2 (62.2,88.1) | 8 |
| **Week 24** | 86.2 (71.6,100) | 8 |

**Table H. Area Under the curve (AUC) values for Xpert direct Ct (Ha), delta Ct (Hb) and percent closing Ct (Hc) ROC curves using treatment failure status at the end of the therapy**

1. **WEEK 4 DATA**

|  | Week 4 Outcomes  (n=91) | | Week 24 Outcomes  (n=91) | |
| --- | --- | --- | --- | --- |
|  | Odds Ratios (95% CI) | p-values | Odds Ratios  (95% CI) | p-values |
| **Model 1:** |  |  |  |  |
| Xpert Ct baseline | 1.12  (1.01, 1.25) | 0.028 | 1.16  (0.97, 1.38) | 0.115 |
| Delta Ct week 4 | 1.16  (1.05, 1.27) | 0.002 | 1.06  (0.93, 1.21) | 0.407 |
| **Model 2:** |  |  |  |  |
| Xpert Ct baseline | 1.03  (0.93, 1.15) | 0.580 | 1.15  (0.96, 1.37) | 0.130 |
| Percent closing week 4 | 122.76  (8.96, 1681.4) | <0.001 | 3.65  (0.29, 45.6) | 0.314 |

1. **WEEK 8 DATA.**

|  | Week 8 Outcomes  (n=91) | | Week 24 Outcomes  (n=91) | |
| --- | --- | --- | --- | --- |
|  | Odds Ratios  (95% CI) | p-values | Odds Ratios  (95% CI) | p-values |
| **Model 1:** |  |  |  |  |
| Xpert Ct baseline | 1.31  (1.15, 1.49) | <0.001 | 1.24  (1.03, 1.50) | 0.024 |
| Delta Ct week 8 | 1.22  (1.12, 1.35) | <0.001 | 1.15  (1.00, 1.31) | 0.050 |
| **Model 2:** |  |  |  |  |
| Xpert Ct baseline | 1.20  (1.07, 1.35) | 0.002 | 1.21  (1.00, 1.46) | 0.045 |
| Percent closing week 8 | 29.42  (5.42, 159.75) | <0.001 | 8.69  (1.03, 73.3) | 0.047 |

1. **WEEK 24 DATA**

|  | Week 24 Outcomes  (n=91) | |
| --- | --- | --- |
|  | Odds Ratios (95% CI) | p-values |
| **Model 1:** |  |  |
| Xpert Ct baseline | 1.36 (1.10, 1.68) | 0.004 |
| Delta Ct week 24 | 1.32 (1.12, 156) | 0.001 |
| **Model 2:** |  |  |
| Xpert Ct baseline | 1.25 (1.03, 1.54) | 0.024 |
| Percent closing week 24 | 149.35 (7.21, 3091.72) | 0.001 |

**Table I. Multiple variable logistic regression models**

**Supplementary References**

1. Wallis RS, Doherty TM, Onyebujoh P, Vahedi M, Laang H, Olesen O, et al. Biomarkers for tuberculosis disease activity, cure, and relapse. Lancet Infect Dis. 2009;9(3):162-72.

2. Mitchison DA. Assessment of new sterilizing drugs for treating pulmonary tuberculosis by culture at 2 months. Am Rev Respir Dis. 1993;147(4):1062-3.

3. Wallis RS, Wang C, Doherty TM, Onyebujoh P, Vahedi M, Laang H, et al. Biomarkers for tuberculosis disease activity, cure, and relapse. Lancet Infect Dis. 2010;10(2):68-9.
